# Implementing an Innovative Prehospital Care Provider Training Course in Nine Cambodian Provinces

**DOI:** 10.7759/cureus.656

**Published:** 2016-06-27

**Authors:** Peter Acker, Jennifer A Newberry, Leonard (Bud) F Hattaway, Phan Socheat, Prak P Raingsey, Matthew C Strehlow

**Affiliations:** 1 Department of Emergency Medicine, Stanford University School of Medicine; 2 EMS- Asia, Medical Teams International; 3 Preventive Medicine Department, Cambodian Ministry of Health

**Keywords:** emergency medical services, prehospital care, cambodia, low resource settings, global health

## Abstract

Despite significant improvements in health outcomes nationally, many Cambodians continue to experience morbidity and mortality due to inadequate access to quality emergency medical services. Over recent decades, the Cambodian healthcare system and civil infrastructure have advanced markedly and now possess many of the components required to establish a well functioning emergency medical system. These components include enhanced access to emergency transportation through large scale road development efforts, widspread availability of emergency communication channels via the spread of cellphone and internet technology, and increased access to health services for poor patients through the implementation of health financing schemes. However, the system still lacks a number of key elements, one of which is trained prehospital care providers. Working in partnership with local providers, our team created an innovative, Cambodia-specific prehospital care provider training course to help fill this gap. Participants received training on prehospital care skills and knowledge most applicable to the Cambodian healthcare system, which was divided into four modules: Basic Prehospital Care Skills and Adult Medical Emergencies, Traumatic Emergencies, Obstetric Emergencies, and Neonatal/Pediatric Emergencies. The course was implemented in nine of Cambodia’s most populous provinces, concurrent with a number of overarching emergency medical service system improvement efforts. Overall, the course was administered to 1,083 Cambodian providers during a 27-month period, with 947 attending the entire course and passing the course completion exam.

## Introduction

Cambodia has made remarkable progress in a number of health outcomes over the past decades. Life expectancy at birth for males and females has increased by over a decade while the maternal mortality ratio and under-five mortality rates have fallen dramatically [1]. Cambodia achieved its Millennium Development Goals for both of these measures but will need new strategies to achieve the Sustainable Development Goals, not only in maternal and child health but also for road traffic injuries and other non-communicable diseases [2]. Improving access to quality emergency medical services (EMS) will be critical to this effort.

Research has shown that implementation of an EMS system can have a positive impact on outcomes for many types of patients across low-, middle- and high-income countries [3-5]. In order to realize these benefits, however, a number of key components must be established and coordinated. Communication systems must be in place to access EMS, trained personnel must be available to provide quality prehospital care, reliable transportation must be accessible, and facilities must be equipped and staffed to receive critically ill patients [6-7].

Although EMS development in Cambodia is in its infancy, much of the infrastructure required to create a well-functioning system already exists. First, mobile communication technology is spreading rapidly. With 94% of Cambodians owning cell phones, and nearly one-third having internet access, technology is creating new opportunities to improve emergency communication systems [8]. Patients and family members can now more readily call for help during emergencies, while many facilities are creating emergency hotlines to streamline the process of accessing emergency care. Second, the transportation infrastructure is strengthened. With the support of foreign aid, the road system in Cambodia has progressed markedly, making ambulance transport safer and faster with improved reach into rural areas [9]. Third, emergency medical care and transport also are becoming more affordable for the poorest patients, with health finance programs being scaled up throughout the country [10]. One of the remaining barriers to the provision of quality EMS is a lack of trained providers.

Up to this point, Cambodia has not had any widespread, coordinated prehospital care provider training programs. Prior training efforts thus far have been limited in scope and reach. However, the concept has been successfully trialed in the country. Husum, et al. were able to show a significant absolute risk reduction in trauma-related mortality (40% to 14.9%) in three rural, landmine-infested districts of Cambodia by creating a network of first responders and providing them with intensive trauma and emergency care training [11]. Steinholt and colleagues extended the Cambodian first responder training to include emergency obstetric care training and noted an associated absolute risk reduction in maternal mortality rate and perinatal mortality rate of 85% and > 80%, respectively (Steinholt M, Chandy H, Ha SO, et al.: Traditional birth attendants; do they make a difference? Abstract presented at the 9^th^ European Congress on Tropical Medicine and International Health, September 6-10, 2015, Basel, Switzerland.).

In hopes of addressing this critical gap in the Cambodian healthcare workforce, Stanford Emergency Medicine International (SEMI), Medical Teams International (MTI), and University Research Co., LLC, partnered together as part of the USAID-funded Quality Health Services Project to create and implement an innovative, Cambodia-specific prehospital care provider training course. This report will describe the intervention, demonstrating its feasibility in nine Cambodian provinces.

## Technical report

### Setting

Cambodia is a predominantly rural Southeast Asian nation of 15.3 million people [12]. Cambodia is considered a low-income country with a gross national income (GNI) per capita of $1,020, ranking 184^th^ of the 214 countries and economies recognized by the World Bank [13]. Currently, Cambodia’s health care expenditure per capita is $61, exceeding the $44 the World Health Organization estimates as the minimum required per person per year to provide basic, life-saving services [14]. At the end of the Khmer Rouge era, Cambodia’s health outcomes were some of the worst in the world. Since that time, marked improvement has been made. Although the infant mortality rate (IMR) and under-5 mortality rate (U5MR) still rank 145^th^ of the 229 countries and economies reporting in 2015 (24.6/1,000 live births and 29/1,000 live births, respectively), and the maternal mortality ratio (MMR) ranks 151^st^ of the 220 countries and economies reporting in 2015 (161/100,000 live births), Cambodia ranks well when compared to other low-income and even low-middle income countries [13]. Cambodia met its Millennium Development Goals early for all three of these metrics (CMDGs). However, Cambodia has yet to announce a strategy to meet the new Sustainable Development Goals to reduce IMR, U5MR, MMR, mortality from road traffic accidents, or premature death from non-communicable diseases, such as cardiovascular conditions.

Cambodia is made up of 25 individual provinces, with each province being further divided into operational districts (OD), such that each OD has a population of between 100,000 and 200,000 residents. Each operational district contains a number of health centers, each serving a catchment area of approximately 10,000 to 20,000 residents. The health centers are typically staffed by nurses and midwives, providing a variety of primary care services, antenatal care, basic delivery services, and postpartum care. Each operational district also contains one government run public referral hospital staffed by nurses, midwives, and doctors. The capacity of these referral hospitals varies. Most are able to provide some basic emergency and inpatient services, while some have access to a blood bank and surgical services. One of the referral hospitals in each province is designated as the provincial referral hospital and is equipped to provide the highest level of care available in the province [15].

Currently, no formal EMS system exists in Cambodia. Ambulances are mostly controlled by and stationed at the government referral hospitals with limited coordination occurring between facilities. Consequently, patients with emergency conditions most commonly arrive at health facilities via private vehicle, motorbike, taxi, or tuk-tuk (a passenger carrying cart pulled by a motorcycle) [16]. Research on interfacility transfer (IFT) for emergency obstetric and neonatal care (EmONC) has shown that nearly half of patients being referred are required to arrange their own transportation. The patients who are transported in a facility-associated vehicle most often do so without the accompaniment of a health care provider (MBS Research Team: Review of the Cambodian Emergency Obstetric and Newborn Care Improvement Plan (2010-2015). Bunsoth M (ed): Cambodian Ministry of Health, Phnom Penh; 2015, unpublished resource). For the fortunate minority of patients who are accompanied by a provider during referral, the provider is typically a staff member from the emergency department or appropriate ward (pediatrics, maternity, etc.) and likely has no training in the provision of ambulance care. There is a lack of data on IFT for other patient populations, such as those suffering trauma or sepsis; however, EmONC in Cambodia has received significant attention and funding in recent years, and thus, these experiences likely represent the best-case scenario [10].

No formal EMS curriculum is offered through any of the health science educational institutes in Cambodia and no government regulation exists. There have been a number of efforts to establish EMS led by international non-governmental organizations (NGO), the Ministry of Health (MOH), and local health facilities. However, these groups faced significant challenges, including difficulty adapting training materials to the Cambodian scenario, inconsistent foreign funding, and failure to integrate their programs into the greater Cambodian healthcare system [17]. Thus far, none has attained widespread acceptance, impact, or sustainability.

### Trainee selection

Cambodia’s health workforce is very limited. While the average European Union nation has nearly four physicians and 7.6 nurses and midwives per 1,000 people, Cambodia possesses a mere 0.2 physicians and only 0.8 nurses and midwives per 1,000 people [13]. This group is constantly depleted by the appeal of higher paying jobs abroad, lucrative private industry positions, and the draw of respected non-clinical postings within the government. In this setting, it seemed infeasible to follow prior foreign EMS models and create a group of providers dedicated solely to prehospital care in Cambodia (i.e., emergency medical technicians or paramedics).

In the current Cambodian system, when a provider is called upon to accompany a patient during transport, the provider is selected from the on-duty staff of the most appropriate ward, depending on the patient type and condition. It was decided that the selection of attendees for the prehospital care training course would follow the same model, selecting attendees from the pool of actively practicing providers at ambulance-possessing government referral hospitals. By task shifting the provision of prehospital care to already employed facility-based providers, the system minimizes staffing needs while also capitalizing on the staff’s preexisting skill sets.

Trainees were invited from all of the 42 referral hospitals in the nine USAID Quality Health Services Project target provinces (Banteay Meanchey, Battambang, Kampong Cham, Kampong Speu, Pailin, Prey Veng, Pursat, Siem Reap, and Tbong Khmum). Participants were selected from the emergency department, maternity ward, and pediatric ward at each included facility and were a mix of physicians, medical assistants, nurses, and midwives. Trainees were selected through a close collaboration with hospital leadership. Trainees were identified as those who had expressed interest and dedication to the provision of quality emergency care and were also the most likely to be involved in emergency patient transportation in the future. Verbal consent was obtained from all participants.

### Training material development

The training materials were created specifically for this course by a team of emergency medicine physicians with expertise in education globally and prehospital care from SEMI as well as a group of prehospital care providers and educators with years of experience working in Cambodia from MTI. The materials were based on a variety of pertinent international guidelines, MOH-approved Cambodian Practice Guidelines, and the team’s prior education experience in Cambodia and other low- and middle-income countries (LMICs). All materials were designed to emphasize the emergency medicine approach to patient care, as this style of practice is unfamiliar to the majority of Cambodian practitioners whose duties often focus on outpatient care and the care of stable inpatients. This approach involves a rapid patient assessment focused on identifying potential life threats and immediate interventions targeted at the abnormalities found on the initial assessment. 

Every attempt was made to adapt the training materials to the Cambodian scenario. First, the training curriculum’s focus was based on the most common emergency conditions encountered by Cambodian providers. Data from prior studies on patterns of emergency patient presentations [16] and the MOH's Health Information System [18] were used to identify 38 high-yield emergency care learning topics. Please see Table 1 for a description of the included topics. Second, all medications recommended were cross-referenced with the MOH’s Essential Medication List [19]. Third, as noted above, the audience was chosen specifically to fit within the constraints of the current Cambodian health care system. Fourth, the skills taught were tailored to ensure they were within the MOH’s scope of practice guidelines for each particular provider group in attendance [15]. Fifth, wherever possible, the visual media items used were obtained in Cambodia to reflect the Cambodian experience. Lastly, all written materials were translated into Khmer and all teaching occurred in the local language.

**Table 1:** Training Topics by Block

Block 1: Basic Prehospital Care Skills and Adult Medical Emergencies	
Introduction to Training General Impression Airway Breathing Circulation Cardiopulmonary Resuscitation Shock Chest Pain	Respiratory Distress Abdominal Pain Snake Bite Altered Mental Status Hypoglycemia Stroke Heat Illness Patient Care Report Use
Block 2: Traumatic Emergencies	
Approach to Trauma Patients Head Trauma Abdominal Trauma Extremity Trauma Pelvic Trauma	Wound Management Smoke Inhalation Burns Lifting and Moving Immobilization
Block 3: Obstetric Emergencies	
Rapid OB Assessment Normal Childbirth Neonatal Resuscitation Multiple Births	Malpresentations Post-Partum Hemorrhage Hypertension in Pregnancy
Block 4: Neonatal and Pediatric Emergencies	
Identifying Ill Pediatric Patients Pediatric Airway and Breathing	Pediatric Circulation Pediatric Coma Pediatric Convulsions

Each topic was presented as an interactive lecture accompanied by either a related case discussion or practical skills station. The lectures were short (15-30 minutes) and focused, highlighting only essential, clinically relevant learning pearls. The case discussions and skills stations were structured to reinforce the approach outlined in the lectures through practice. Facilitators used critical action checklists to assess participants during the cases and skills stations, enabling them to provide consistent, structured feedback. Please see Table 2 for a list of case discussion and skills station topics.

**Table 2:** Case Discussion and Skills Station Topics by Block

Block 1: Basic Prehospital Care Skills and Adult Medical Emergencies	
Case Discussion Topics Shock 75-year-old female with fever Abdominal Pain 24-year-old female with lower abdominal pain and dizziness Chest Pain 65-year-old man with chest pressure Respiratory Distress 48-year-old man with cough and wheezing Altered Mental Status-Hypoglycemia 28-year-old man with progressive confusion Stroke 77-year-old female with slurred speech and right-sided arm weakness Heat Illness 35-year-old male, found outdoors confused and disoriented Snake Bite 18-year-old male who was bit by a snake while working in a field	Skills Stations Topics Airway Adjunct Use Bag Mask Ventilation Manual Airway Maneuvers Airway Suctioning Cardiopulmonary Resuscitation Glucometer Use
Block 2: Traumatic Emergencies	
Case Discussion Topics Abdominal Trauma 42-year-old male bicyclist who was hit by a car Head Trauma 36-year-old female driving a motor scooter, hit by a car Chest Trauma 50-year-old male pedestrian struck by a truck Extremity Trauma 18-year-old female fell off a motor scooter with pain to right arm Pelvic Trauma 65-year-old female pedestrian who was struck by a car Immobilization 48-year-old unrestrained driver involved in a collision Smoke Inhalation and Burns 23-year-old male who was trapped in a burning building Wound Management 35-year-old male cut himself with a machete while working in the fields	Skills Stations Topics Open Chest Wound Treatment Extremity Hemorrhage Control Extremity Injury Skills: Splinting and Sling Application Pelvic Binder Application Spinal Immobilization
Block 3: Obstetric Emergencies	
Case Discussion Topics Neonatal Resuscitation Neonate immediately after delivery with bradycardia Normal Childbirth and Post-Delivery Care 24-year-old female in active labor Post-Partum Hemorrhage 25-year-old female with heavy vaginal bleeding after delivery Preeclampsia 24-year-old pregnant female with progressive headache and blurred vision	Skills Stations Topics Breech Delivery Cord Presentation Management Limb Presentation Management Intramuscular Oxytocin Administration Intravenous Oxytocin Administration Magnesium Sulfate Administration Non-Pneumatic Anti-Shock Garment (NASG) Application Aortic Compression in Post-Partum Hemorrhage Normal Childbirth Management Shoulder Dystocia Management
Block 4: Neonatal and Pediatric Emergencies	
Case Discussion Topics Pediatric Convulsions 3-year-old boy, unresponsive with rhythmic jerking of upper and lower extremities Pediatric Hypoglycemia 1-year-old boy with witnessed convulsion, now unconscious Pediatric Respiratory Distress 4-month-old female with rapid breathing Pediatric Shock 7-month-old male with lethargy after 2 days of diarrhea and poor oral intake Pediatric Shock with Severe Acute Malnutrition 15-month-old female with lethargy after 2 days of diarrhea and poor oral intake	Skills Stations Topics Neonatal Bag Mask Ventilation Neonatal Cardiopulmonary Resuscitation Pediatric Airway Adjunct Use Pediatric Choking Management Pediatric Manual Airway Maneuvers Medication Nebulization Glucose Administration

**Figure 1:**
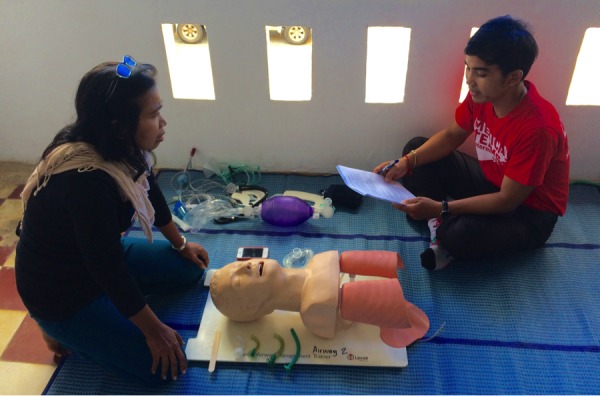
Photo of Training Course instructor providing feedback to a participant on their performance during a clinical skills scenario.

Student learning was assessed in two ways. Knowledge improvement was measured through a comparison of a 25 multiple choice question pre- and post-test scores. Students also took part in two structured clinical skills scenarios with a variety of themes, including airway management, bag mask ventilation, normal childbirth, and neonatal resuscitation. The clinical skills scenarios were graded using critical action checklists. Figure 1 shows an instructor providing feedback on a clinical skills scenario to a participant using a critical action checklist.

Input and revisions to training and testing materials were sought and incorporated in an iterative fashion. Cambodian and international experts in the fields of trauma, pediatrics, neonatal care, and obstetric emergencies were consulted and provided guidance to SEMI during the materials creation process. Once drafted, the materials were assessed by MTI’s team of facilitator’s for their applicability in the Cambodian health system and to ensure the level of the content was appropriate for the intended audience. During the rollout phase, further feedback was sought at each stage of the training from recruited trainers and training participants and, when appropriate, incorporated into the teaching materials prior to further dissemination.

### Training implementation

The 38 training topics were grouped thematically into four modules: Basic Prehospital Care Skills and Adult Medical Emergencies, Traumatic Emergencies, Obstetric Emergencies, and Neonatal/Pediatric Emergencies. The four modules were delivered over two 3.5-day blocks, resulting in a 56-hour, Cambodia-specific, prehospital care provider training course.

Each block of training was initially rolled out by MTI’s facilitators to groups of master trainers during the Training of Trainer (TOT) sessions at key provincial hubs. Working alongside MTI’s facilitator team and local content experts, the master trainers then administered both blocks of training in their home regions. Due to scheduling logistics secondary to limited field staff, the two training blocks were separated by six to nine month's time. To improve knowledge retention, a review of the previous training materials was incorporated into the second block’s content. 

Over a 27-month period (December 2013-February 2016), four TOTs were performed, training 64 regional master trainers. These trainers, in conjunction with MTI facilitators, then provided 58 training courses to 1,083 providers throughout the nine target provinces. Of the 1,083 providers trained, 947 (87.4%) attended both blocks of training courses and received a certificate of completion for passing the course completion exam. Of the 136 students who did not complete the training, the majority were unable to do so due to scheduling conflicts that prevented them from attending the second block of training courses.

## Discussion

Prior studies from developed and developing countries have shown that the institution of an emergency medical system can have positive impacts on morbidity and mortality outcomes [3-5, 20]. The potential impact of an EMS system is likely to be even more pronounced in Cambodia, a country in which 80% of the population resides in rural areas and may face prolonged travel time to and between health facilities. However, the full benefits of the system will not be realized until patients are able to receive care from well-trained prehospital care providers during transport.

This course utilized a number of strategies to maximize potential impact. First, it had the support of the MOH. The MOH approved the training manual upon which much of the training curriculum was based. MOH staff were also trained as master trainers and were involved in rolling the curriculum out to the nine provinces. The MOH buy-in lent the program credibility and helped garner support from the staff and leadership at the various referral hospitals involved. The project partners had initially gained the support of the MOH during their prior work in Cambodia and have continued to work closely with the MOH during the ongoing referral and EMS system development efforts.

Second, the implementing team had extensive experience with the Cambodian medical system, understanding its structure well enough to adapt the proposed prehospital care provider training to fit. From prior experience, it was clear that creating a cadre of ambulance-only providers, as are utilized in many other countries, would not be feasible due to financial constraints and limited healthcare workforce. Instead, potential ambulance providers would need to be picked from currently employed hospital staff and would be cross-trained to perform both duties.

Third, the training courses were targeted at the providers most likely to utilize the skills being taught. With the majority of Cambodian ambulances being health facility-based, the trainees were accordingly selected only from ambulance-equipped facilities. They were also chosen by facility leadership based on their likelihood of involvement in ambulance patient transport. 

**Figure 2:**
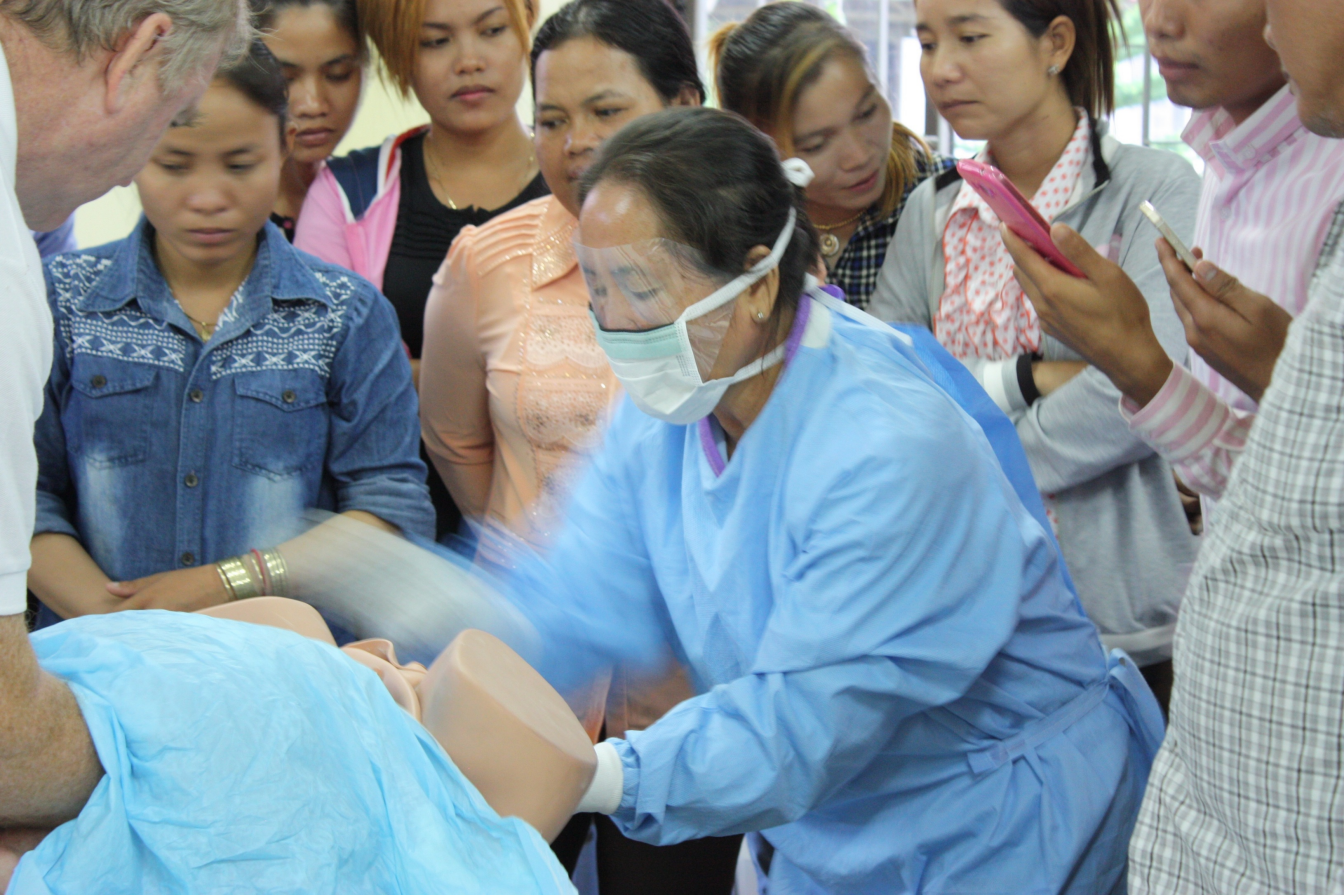
Photo of Training Government facilitator demonstrating delivery techniques to participants.

Fourth, the course content was tailored to the most likely scenarios the providers would face, based on existing data on patterns of emergency presentations and referrals [16]. With obstetric and peripartum conditions comprising a large portion of hospital presentations in Cambodia, maternal and pediatric emergencies were given more attention in this curriculum than in most prehospital care provider training curriculums from more developed EMS systems. Figure 2 captures a government facilitator demonstrating breech delivery procedures to training participants.

Fifth, the course content was simple and utilitarian. The providers involved in the course had healthcare backgrounds but lacked prehospital and emergency care skills. With this in mind, the content was created to capitalize on their prior knowledge, while adding to it the emergency assessment and intervention skills required to provide effective prehospital care. The lectures were short and focused on enhancing information acquisition. The content was practically oriented and highlighted the approach to the treatment of emergency patients and associated procedures, forgoing the background anatomy and physiology often included in prehospital care provider training courses. The lectures were paired with case discussions and scenario-based skill stations to reinforce the learned concepts through spaced repetition and application.

While the initial training phase has been completed, ongoing follow-up education and evaluation of the trainees continues. The focus of future programming will be on continued prehospital care skill development and the provision of refresher education.

The course did experience a number of limitations. First, due to a limited quantity of MTI field facilitators and a large number of training sites, there were significant time intervals between Block 1 and Block 2 of the training curriculum. Although skills and information from the first course were reinforced during the second training, some of the knowledge was likely lost in the interim period. 

Second, as mentioned above, 136 students who attended the first block of training were not able to attend the second. These students missed key prehospital care skills and information, particularly focused on maternal and pediatric emergencies. Most of these absences were due to scheduling conflicts at their home facilities and could likely have been overcome by working more closely with hospital leadership to ensure schedules were coordinated effectively. 

Third, administration of the written and skills exams was inconsistent. Throughout the course of the training, a number of versions of the written pre- and post-test were used. There was also variability in the application of grading criteria for the clinical scenarios. Although course instructors were taught to teach, little time was spent teaching them how to evaluate their students; thus, practices varied. In the future, strengthening the evaluation component of the course will be a priority.

Fourth, assessing the impact of the course on the clinical practice of its graduates has been difficult. Although it would be optimal to have data on the frequency and appropriateness with which the taught skills were utilized, the regularity with which graduates of the course were involved in ambulance transports, and the outcomes of patients being transported by course graduates, this data is not yet available. However, as the quality of emergency transport and referral data improves, this type of information will likely become accessible. In the interim, surrogate markers for impact on practice are being collected during follow-up visits. Provider practice habits are being assessed through interviews while the retention of prehospital skills is being tested through further graded clinical skill scenarios.

Although the course itself worked to overcome one set of challenges facing the development of EMS in Cambodia, our group was addressing a number of other issues concurrently. Preliminary survey work found that the majority of Cambodian ambulances lacked the equipment necessary to provide many essential prehospital interventions. During the training period, a minimum standard ambulance equipment list was prepared by the project partners, approved by the MOH, and distributed to referral hospital leadership to advocate for more appropriate equipment on their ambulances. It was also found that referral communication, particularly between ambulance providers and receiving facility staff, was minimal. During this project, a standardized patient care report (PCR) form was drafted by the project partners, approved by the MOH for nationwide use, and is currently being distributed to all referral hospitals throughout Cambodia. 

While improving prehospital care provider ability is one means of improving access to quality emergency care in Cambodia, there are many other challenges in need of attention. Further efforts are required to strengthen referral communication strategies, streamlining the process through which referring providers contact receiving providers to alert them of pending referrals, gather treatment guidance, and arrange transfer logistics. Ambulance coordination systems must be improved to decrease delays in care and guarantee that referred patients are delivered to appropriate receiving facilities in a timely manner. Lastly, hospital-based emergency care provider capacity must be enhanced to ensure that referred patients receive high-quality care upon arrival at receiving facilities. 

## Conclusions

Establishment of a functioning EMS system is a vital step on the path towards improving health outcomes for emergently ill and injured patients in Cambodia. The training described utilized an innovative, streamlined, Cambodia-specific curriculum to create a cadre of trained prehospital care providers. These providers fill a critical gap in the Cambodian health workforce and address a significant barrier to the provision of quality prehospital care. This capacity building effort, in conjunction with broader referral system strengthening efforts, has the potential to bring a significant, sustainable change to the Cambodian emergency care and referral system. 
